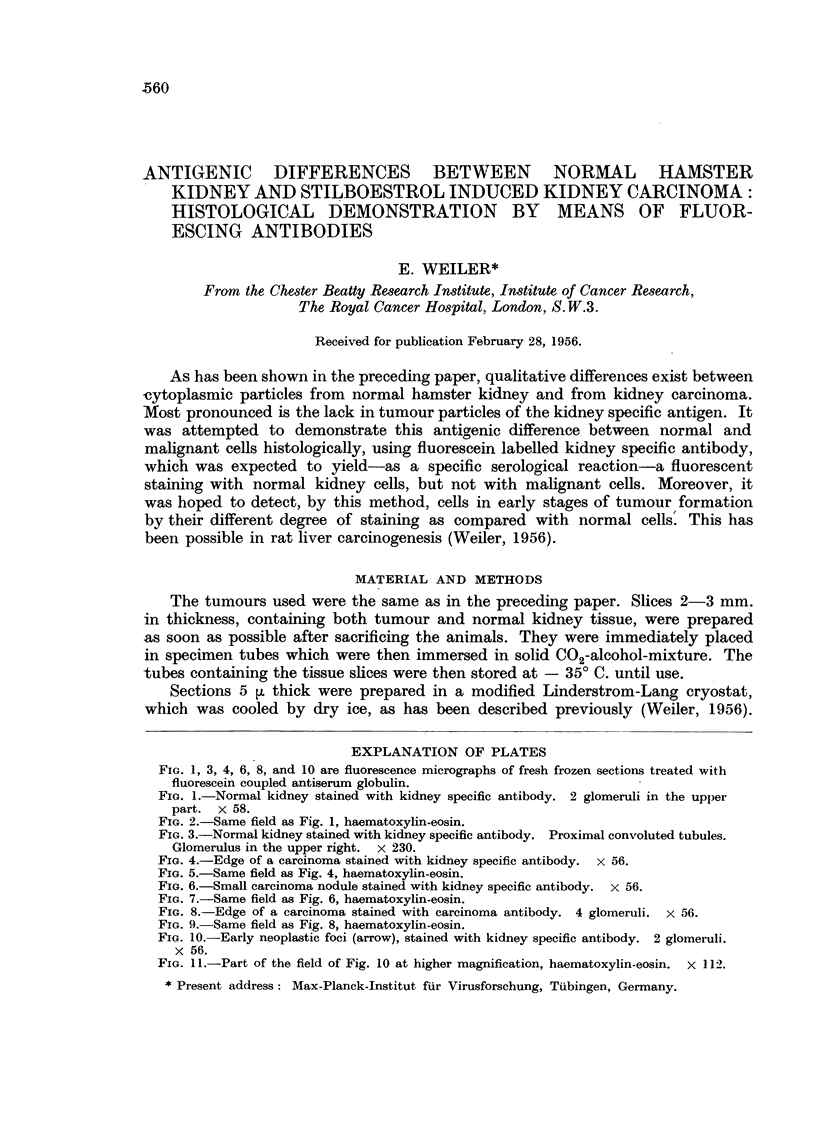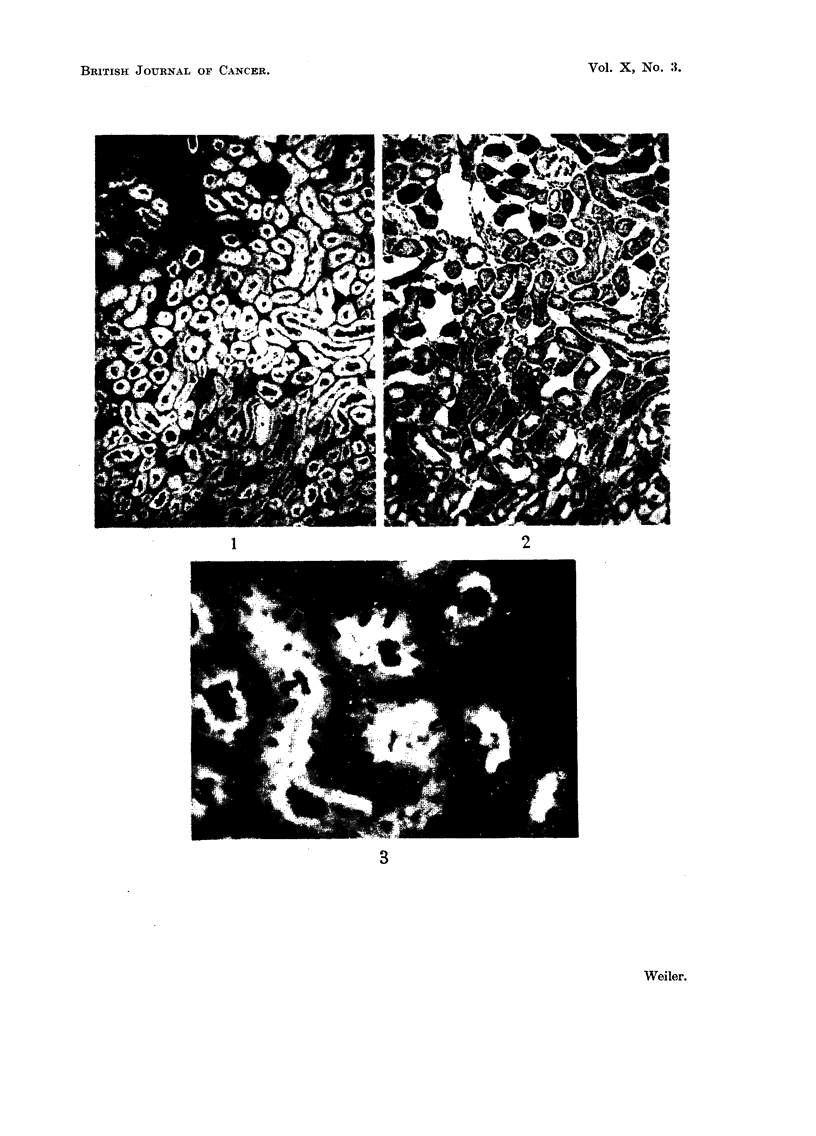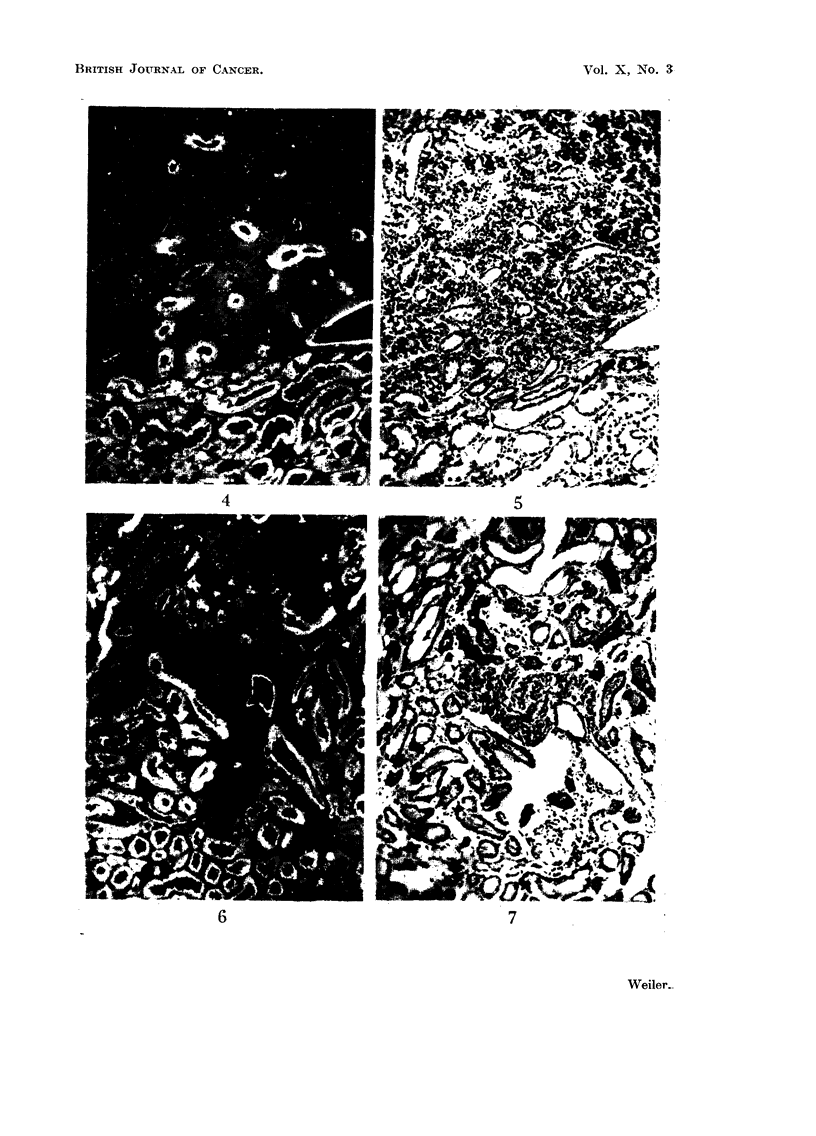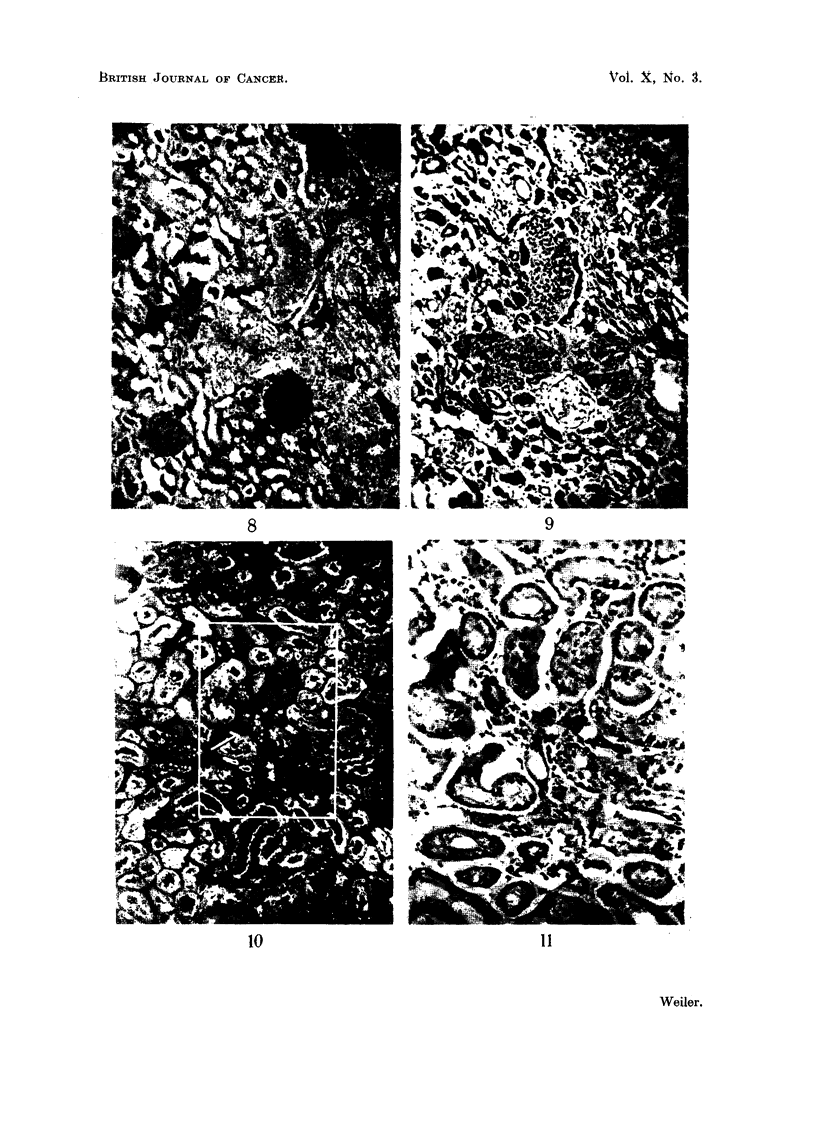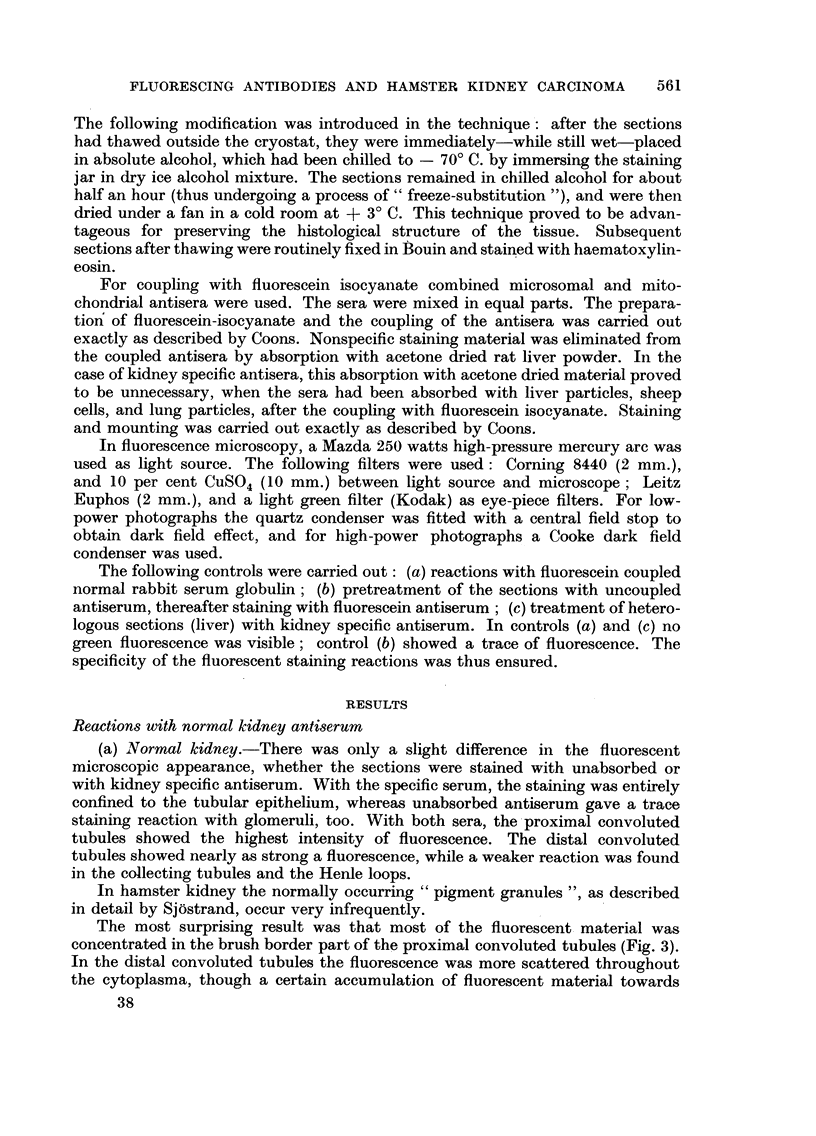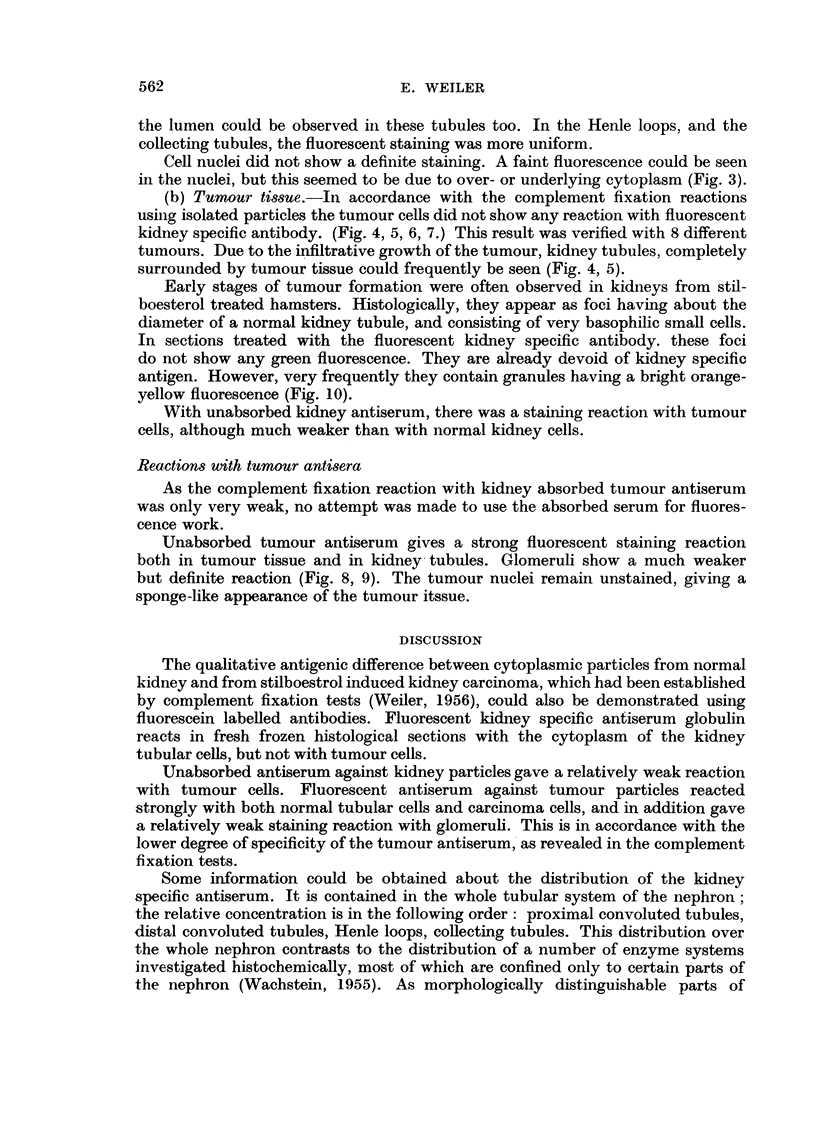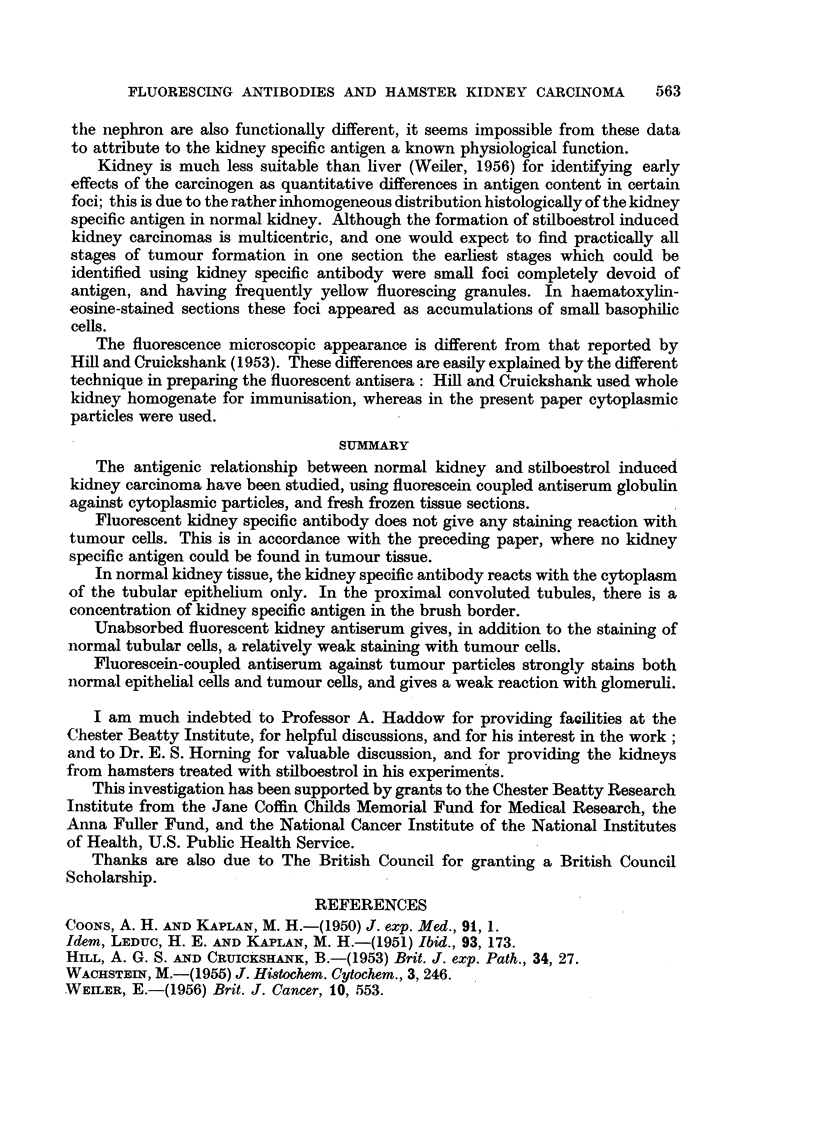# Antigenic Differences between Normal Hamster Kidney and Stilboestrol Induced Kidney Carcinoma: Histological Demonstration by Means of Fluorescing Antibodies

**DOI:** 10.1038/bjc.1956.66

**Published:** 1956-09

**Authors:** E. Weiler

## Abstract

**Images:**


### ANTIGENIC DIFFERENCES BETWEEN NORMAL HAMSTER KIDNEY AND STILBOESTROL INDUCED KIDNEY CARCINOMA: HISTOLOGICAL DEMONSTRATION BY MEANS OF FLUORESCING ANTIBODIES

E. WEILER*

*From the Chester Beatty Research Institute, Institute of Cancer Research, The Royal Cancer Hospital, London, S.W.3.*

Received for publication February 28, 1956.

As has been shown in the preceding paper, qualitative differences exist between cytoplasmic particles from normal hamster kidney and from kidney carcinoma. Most pronounced is the lack in tumour particles of the kidney specific antigen. It was attempted to demonstrate this antigenic difference between normal and malignant cells histologically, using fluorescein labelled kidney specific antibody, which was expected to yield—as a specific serological reaction—a fluorescent staining with normal kidney cells, but not with malignant cells. Moreover, it was hoped to detect, by this method, cells in early stages of tumour formation by their different degree of staining as compared with normal cells. This has been possible in rat liver carcinogenesis (Weiler, 1956).

### MATERIAL AND METHODS

The tumours used were the same as in the preceding paper. Slices 2—3 mm. in thickness, containing both tumour and normal kidney tissue, were prepared as soon as possible after sacrificing the animals. They were immediately placed in specimen tubes which were then immersed in solid $CO_2$-alcohol-mixture. The tubes containing the tissue slices were then stored at — 35° C. until use.

Sections 5 $\mu$ thick were prepared in a modified Linderstrom-Lang cryostat, which was cooled by dry ice, as has been described previously (Weiler, 1956).

---

### EXPLANATION OF PLATES

FIG. 1, 3, 4, 6, 8, and 10 are fluorescence micrographs of fresh frozen sections treated with fluorescein coupled antiserum globulin.

FIG. 1.—Normal kidney stained with kidney specific antibody. 2 glomeruli in the upper part. × 58.

FIG. 2.—Same field as Fig. 1, haematoxylin-eosin.

FIG. 3.—Normal kidney stained with kidney specific antibody. Proximal convoluted tubules. Glomerulus in the upper right. × 230.

FIG. 4.—Edge of a carcinoma stained with kidney specific antibody. × 56.

FIG. 5.—Same field as Fig. 4, haematoxylin-eosin.

FIG. 6.—Small carcinoma nodule stained with kidney specific antibody. × 56.

FIG. 7.—Same field as Fig. 6, haematoxylin-eosin.

FIG. 8.—Edge of a carcinoma stained with carcinoma antibody. 4 glomeruli. × 56.

FIG. 9.—Same field as Fig. 8, haematoxylin-eosin.

FIG. 10.—Early neoplastic foci (arrow), stained with kidney specific antibody. 2 glomeruli. × 56.

FIG. 11.—Part of the field of Fig. 10 at higher magnification, haematoxylin-eosin. × 112.

* Present address: Max-Planck-Institut für Virusforschung, Tübingen, Germany.

1

2

3

4

5

6

7

Weiler.

8

9

10

11

Weiler.

The following modification was introduced in the technique: after the sections had thawed outside the cryostat, they were immediately—while still wet—placed in absolute alcohol, which had been chilled to $-70°$ C. by immersing the staining jar in dry ice alcohol mixture. The sections remained in chilled alcohol for about half an hour (thus undergoing a process of "freeze-substitution"), and were then dried under a fan in a cold room at $+3°$ C. This technique proved to be advantageous for preserving the histological structure of the tissue. Subsequent sections after thawing were routinely fixed in Bouin and stained with haematoxylin-eosin.

For coupling with fluorescein isocyanate combined microsomal and mitochondrial antisera were used. The sera were mixed in equal parts. The preparation of fluorescein-isocyanate and the coupling of the antisera was carried out exactly as described by Coons. Nonspecific staining material was eliminated from the coupled antisera by absorption with acetone dried rat liver powder. In the case of kidney specific antisera, this absorption with acetone dried material proved to be unnecessary, when the sera had been absorbed with liver particles, sheep cells, and lung particles, after the coupling with fluorescein isocyanate. Staining and mounting was carried out exactly as described by Coons.

In fluorescence microscopy, a Mazda 250 watts high-pressure mercury arc was used as light source. The following filters were used: Corning 8440 (2 mm.), and 10 per cent $CuSO_4$ (10 mm.) between light source and microscope; Leitz Euphos (2 mm.), and a light green filter (Kodak) as eye-piece filters. For low-power photographs the quartz condenser was fitted with a central field stop to obtain dark field effect, and for high-power photographs a Cooke dark field condenser was used.

The following controls were carried out: (*a*) reactions with fluorescein coupled normal rabbit serum globulin; (*b*) pretreatment of the sections with uncoupled antiserum, thereafter staining with fluorescein antiserum; (*c*) treatment of heterologous sections (liver) with kidney specific antiserum. In controls (*a*) and (*c*) no green fluorescence was visible; control (*b*) showed a trace of fluorescence. The specificity of the fluorescent staining reactions was thus ensured.

### RESULTS

### *Reactions with normal kidney antiserum*

(a) *Normal kidney.*—There was only a slight difference in the fluorescent microscopic appearance, whether the sections were stained with unabsorbed or with kidney specific antiserum. With the specific serum, the staining was entirely confined to the tubular epithelium, whereas unabsorbed antiserum gave a trace staining reaction with glomeruli, too. With both sera, the proximal convoluted tubules showed the highest intensity of fluorescence. The distal convoluted tubules showed nearly as strong a fluorescence, while a weaker reaction was found in the collecting tubules and the Henle loops.

In hamster kidney the normally occurring "pigment granules", as described in detail by Sjöstrand, occur very infrequently.

The most surprising result was that most of the fluorescent material was concentrated in the brush border part of the proximal convoluted tubules (Fig. 3). In the distal convoluted tubules the fluorescence was more scattered throughout the cytoplasma, though a certain accumulation of fluorescent material towards the lumen could be observed in these tubules too. In the Henle loops, and the collecting tubules, the fluorescent staining was more uniform.

Cell nuclei did not show a definite staining. A faint fluorescence could be seen in the nuclei, but this seemed to be due to over- or underlying cytoplasm (Fig. 3).

(b) *Tumour tissue.*—In accordance with the complement fixation reactions using isolated particles the tumour cells did not show any reaction with fluorescent kidney specific antibody. (Fig. 4, 5, 6, 7.) This result was verified with 8 different tumours. Due to the infiltrative growth of the tumour, kidney tubules, completely surrounded by tumour tissue could frequently be seen (Fig. 4, 5).

Early stages of tumour formation were often observed in kidneys from stilboesterol treated hamsters. Histologically, they appear as foci having about the diameter of a normal kidney tubule, and consisting of very basophilic small cells. In sections treated with the fluorescent kidney specific antibody. these foci do not show any green fluorescence. They are already devoid of kidney specific antigen. However, very frequently they contain granules having a bright orange-yellow fluorescence (Fig. 10).

With unabsorbed kidney antiserum, there was a staining reaction with tumour cells, although much weaker than with normal kidney cells.

*Reactions with tumour antisera*

As the complement fixation reaction with kidney absorbed tumour antiserum was only very weak, no attempt was made to use the absorbed serum for fluorescence work.

Unabsorbed tumour antiserum gives a strong fluorescent staining reaction both in tumour tissue and in kidney tubules. Glomeruli show a much weaker but definite reaction (Fig. 8, 9). The tumour nuclei remain unstained, giving a sponge-like appearance of the tumour itssue.

### DISCUSSION

The qualitative antigenic difference between cytoplasmic particles from normal kidney and from stilboestrol induced kidney carcinoma, which had been established by complement fixation tests (Weiler, 1956), could also be demonstrated using fluorescein labelled antibodies. Fluorescent kidney specific antiserum globulin reacts in fresh frozen histological sections with the cytoplasm of the kidney tubular cells, but not with tumour cells.

Unabsorbed antiserum against kidney particles gave a relatively weak reaction with tumour cells. Fluorescent antiserum against tumour particles reacted strongly with both normal tubular cells and carcinoma cells, and in addition gave a relatively weak staining reaction with glomeruli. This is in accordance with the lower degree of specificity of the tumour antiserum, as revealed in the complement fixation tests.

Some information could be obtained about the distribution of the kidney specific antiserum. It is contained in the whole tubular system of the nephron ; the relative concentration is in the following order : proximal convoluted tubules, distal convoluted tubules, Henle loops, collecting tubules. This distribution over the whole nephron contrasts to the distribution of a number of enzyme systems investigated histochemically, most of which are confined only to certain parts of the nephron (Wachstein, 1955). As morphologically distinguishable parts of the nephron are also functionally different, it seems impossible from these data to attribute to the kidney specific antigen a known physiological function.

Kidney is much less suitable than liver (Weiler, 1956) for identifying early effects of the carcinogen as quantitative differences in antigen content in certain foci; this is due to the rather inhomogeneous distribution histologically of the kidney specific antigen in normal kidney. Although the formation of stilboestrol induced kidney carcinomas is multicentric, and one would expect to find practically all stages of tumour formation in one section the earliest stages which could be identified using kidney specific antibody were small foci completely devoid of antigen, and having frequently yellow fluorescing granules. In haematoxylin-eosine-stained sections these foci appeared as accumulations of small basophilic cells.

The fluorescence microscopic appearance is different from that reported by Hill and Cruickshank (1953). These differences are easily explained by the different technique in preparing the fluorescent antisera : Hill and Cruickshank used whole kidney homogenate for immunisation, whereas in the present paper cytoplasmic particles were used.

### SUMMARY

The antigenic relationship between normal kidney and stilboestrol induced kidney carcinoma have been studied, using fluorescein coupled antiserum globulin against cytoplasmic particles, and fresh frozen tissue sections.

Fluorescent kidney specific antibody does not give any staining reaction with tumour cells. This is in accordance with the preceding paper, where no kidney specific antigen could be found in tumour tissue.

In normal kidney tissue, the kidney specific antibody reacts with the cytoplasm of the tubular epithelium only. In the proximal convoluted tubules, there is a concentration of kidney specific antigen in the brush border.

Unabsorbed fluorescent kidney antiserum gives, in addition to the staining of normal tubular cells, a relatively weak staining with tumour cells.

Fluorescein-coupled antiserum against tumour particles strongly stains both normal epithelial cells and tumour cells, and gives a weak reaction with glomeruli.

I am much indebted to Professor A. Haddow for providing facilities at the Chester Beatty Institute, for helpful discussions, and for his interest in the work ; and to Dr. E. S. Horning for valuable discussion, and for providing the kidneys from hamsters treated with stilboestrol in his experiments.

This investigation has been supported by grants to the Chester Beatty Research Institute from the Jane Coffin Childs Memorial Fund for Medical Research, the Anna Fuller Fund, and the National Cancer Institute of the National Institutes of Health, U.S. Public Health Service.

Thanks are also due to The British Council for granting a British Council Scholarship.

### REFERENCES

COONS, A. H. AND KAPLAN, M. H.—(1950) *J. exp. Med.*, **91**, 1.

*Idem*, LEDUC, H. E. AND KAPLAN, M. H.—(1951) *Ibid.*, **93**, 173.

HILL, A. G. S. AND CRUICKSHANK, B.—(1953) *Brit. J. exp. Path.*, **34**, 27.

WACHSTEIN, M.—(1955) *J. Histochem. Cytochem.*, **3**, 246.

WEILER, E.—(1956) *Brit. J. Cancer*, **10**, 553.